# Individuality counts: A new comprehensive approach to foraging strategies of a tropical marine predator

**DOI:** 10.1007/s00442-021-04850-w

**Published:** 2021-01-24

**Authors:** Jonas F. L. Schwarz, Sina Mews, Eugene J. DeRango, Roland Langrock, Paolo Piedrahita, Diego Páez-Rosas, Oliver Krüger

**Affiliations:** 1 Department of Animal Behaviour, Bielefeld University, Bielefeld, Germany; 2 Department of Business Administration and Economics, Bielefeld University, Bielefeld, Germany; 3 Facultad de Ciencias de La Vida, Escuela Superior Politécnica del Litoral, Guayaquil, Ecuador; 4 Galápagos Science Center, Universidad San Francisco de Quito, Puerto Baquerizo Moreno, Ecuador; 5 Dirección Parque Nacional Galápagos, Unidad Técnica Operativa San Cristóbal, Puerto Baquerizo Moreno, Ecuador

**Keywords:** Galápagos sea lion, Broken stick algorithm, Hidden markov models, Individual differences, Conservation

## Abstract

Foraging strategies are of great ecological interest, as they have a strong impact on the fitness of an individual and can affect its ability to cope with a changing environment. Recent studies on foraging strategies show a higher complexity than previously thought due to intraspecific variability. To reliably identify foraging strategies and describe the different foraging niches they allow individual animals to realize, high-resolution multivariate approaches which consider individual variation are required. Here we dive into the foraging strategies of Galápagos sea lions (*Zalophus wollebaeki*), a tropical predator confronted with substantial annual variation in sea surface temperature. This affects prey abundance, and El Niño events, expected to become more frequent and severe with climate change, are known to have dramatic effects on sea lions. This study used high-resolution measures of depth, GPS position and acceleration collected from 39 lactating sea lion females to analyze their foraging strategies at an unprecedented level of detail using a novel combination of automated broken stick algorithm, hierarchical cluster analysis and individually fitted multivariate hidden Markov models. We found three distinct foraging strategies (pelagic, benthic, and night divers), which differed in their horizontal, vertical and temporal distribution, most likely corresponding to different prey species, and allowed us to formulate hypotheses with regard to adaptive values under different environmental scenarios. We demonstrate the advantages of our multivariate approach and inclusion of individual variation to reliably gain a deeper understanding of the adaptive value and ecological relevance of foraging strategies of marine predators in dynamic environments.

**Supplementary Information:**

The online version contains supplementary material available at 10.1007/s00442-021-04850-w.

## Introduction

Foraging behavior is a key aspect in understanding the ecology of a species, a population, and individuals, as the ability to balance energy intake with energy expenditure during foraging determines the resources that individuals can invest into self-preservation and reproduction (Schoener 1971). The study of foraging behavior is a complex topic, as highly diverse foraging strategies can be found not only between species but also within a species or even within a given population (e.g., Bolnick et al. 2003; Ceia and Ramos 2015; Cleasby et al. 2015). Intraspecific variation in foraging strategies may exist due to several proximate mechanisms and can be closely linked to the growing field of stable individual differences (Sih et al. 2004; Réale et al. 2007). Physiological limitations, such as body size, condition and age, can be factors driving inter-individual behavioral differences (Polis 1894; Skulason and Smith 1995). In marine mammals, for example, larger animals can often store more oxygen in relation to their metabolic rate allowing them to dive deeper and thereby exploit different foraging habitats (Costa et al. 2004). Another driving force behind differences in foraging can be intraspecific competition (Kuhn et al. 2014). This is common in colonial-living species, such as many seabirds and pinniped species, which are constrained to a limited foraging range and may overlap strongly with other conspecifics that exploit similar niches (Kernaléguen et al. 2015).

Differences in foraging strategies can be adaptive, especially under fluctuating environmental conditions. This was demonstrated for bluegill sunfish, *Lepomis macrochirus*, where foraging specialists showed higher foraging success under stable environmental conditions, while generalists were more successful under unstable environmental conditions (Wilson 1998; Wilson and Godin 2009). This example underlines the importance of individual variability for understanding the responses of individuals and populations to environmental change (Violle et al. 2012). Intriguingly, foraging strategies are often found to be quite stable within individuals of many predators (Hoelzel et al. 1989; Lowther et al. 2011; Patrick et al. 2015; McHuron et al. 2018), although a greater flexibility might seem more adaptive to cope with dynamic, fluctuating environmental conditions. Stability may be due to costs associated with a change in foraging strategy and may, therefore, be the best strategy to cope with uncertain environmental conditions (McHuron et al. 2018).

Advances in biologging technology allow a deeper study of foraging behavior in aquatic species like pinnipeds, with recent developments revealing a high complexity of foraging strategies (Baylis et al. 2015). Variation in dive behavior with two or more foraging strategies has been observed in certain otariid species among individuals of the same sex or body size (Chilvers 2008; Lowther et al. 2011; Baylis 2015; McHuron et al. 2016). Studies have also gathered increasing evidence that an individual-specific foraging strategy is often retained across long time spans (Chilvers and Wilkinson 2009; Kernaléguen et al. 2012, 2016; McHuron et al. 2016). Taking the individual variability into account when analyzing foraging strategies will allow for a greater precision and a better understanding of the part of the ecological niche individuals occupy (Bolnick et al. 2003). The need for a better understanding of foraging strategies was highlighted by Chilvers and Wilkinson (2009), who showed a major overlap of one strategy of the endangered New Zealand sea lion with fisheries, leading to a higher death risk for these animals compared with conspecifics pursuing other strategies.

The endangered Galápagos sea lion, *Zalophus wollebaeki*, lives in a challenging, highly variable tropical habitat strongly affected by El Niño-Southern Oscillation events (Trillmich and Limberger 1985) and is subject to increased environmental variation associated with climate change (Trenberth and Hoar 1997). Females nurse their pup for an average of two to three years until independence (Trillmich and Wolf 2008), which makes them dependent on foraging areas near the colony, a factor that increases intraspecific competition (Urquía and Páez-Rosas 2019). Previous studies of Galápagos sea lions identified variability of diving behavior and diversity of targeted prey species (Páez-Rosas and Aurioles-Gamboa 2010; Jeglinski et al. 2015), and different foraging strategies between and also within colonies have been described (Villegas-Amtmann et al. 2008; Villegas-Amtmann and Costa 2010; Páez-Rosas et al. 2017). A better understanding of these strategies and the individual foraging niches is needed, as this will enable predictions about the adaptive value in a rapidly changing environment and may help assess the consequences of future challenges such as climate change. Key element is the identification of reliable characteristics of foraging behavior to design models that predict when and how foraging occurs while recognizing the great individual variability (DeRango and Schwarz 2021).

The aim of this study is to identify and describe foraging strategies of Galápagos sea lion females and their foraging niche in hitherto unmatched detail with the help of a novel combination of advanced dive analysis techniques. Our analytical approach involves three steps: we first identify potential foraging episodes in the dive, then group individuals according to the similarity of their foraging episodes, and finally identify within-group behavioral modes. For the first step, an automated broken stick algorithm and vertical sinuosity measurements are employed to create a novel set of detailed dive variables describing foraging episodes. A hierarchical cluster analysis is applied to these variables to build groups of individuals exhibiting similar foraging behavior to identify foraging strategies. To explore the corresponding foraging niches in more detail, we identify underlying behavioral modes and their dynamics using individually fitted multivariate hidden Markov models. This study thus expands the research on diving behavior of the endangered Galápagos sea lion but also emphasizes the advantages of recognizing individual differences when analyzing foraging behaviors of predators in general. Deeper insight into the foraging ecology of species enables ecological consequences to be studied and can improve conservation management decisions to improve conservation.

## Methods

### Study details

This study was conducted on Caamaño, a small islet in the center of the Galápagos Archipelago near Santa Cruz Island (0° 45′ S, 90° 16′ W). Since 2003, the resident sea lion colony has been part of a long-term monitoring program that includes annual birth and growth assessment of pups, tagging of individuals, and census rounds (see Trillmich et al. 2014), providing detailed life-history data for the majority of individuals.

For the present study, we captured 39 lactating females with hoop-nets, weighed and measured them, and equipped them with a time-depth recorder (MK10, Wildlife Computers, Redmond, WA, USA) on the dorsum behind the shoulder blades (see Jeglinski et al. 2013 for more details). These biologging devices were deployed for approximately two weeks (median 15 days, range 3–22) between October and December of 2018 and 2019. Devices were programmed to record dive depth every 2 s, GPS-position through fastloc-GPS approximately every 4 min, and acceleration data with 32 Hz. The scaled mass index (SMI) as introduced by Peig and Green (2009) was calculated using mass and length data of females taken before the placement of the biologging device (*N* = 37) to measure relative body condition. The age of most females could be identified through the long-term dataset (*N* = 31).

### Dive analysis

**Fig. 1:**
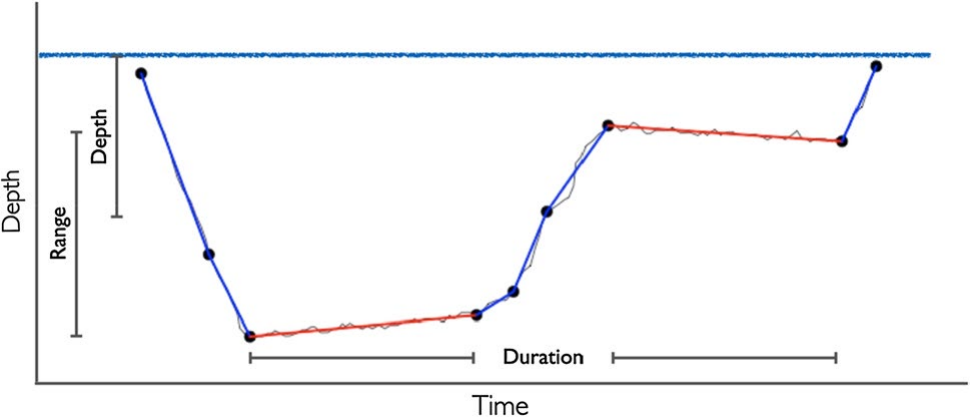
Dive profile analyzed with the broken stick algorithm. Blue segments represent sections with a vertical sinuosity index above 0.9, indicating transit, while red segments represent sections with a vertical sinuosity index below 0.9, indicating foraging episodes

We employed the R package *diveMove* (Luque 2007) to identify dives, using individual zero-offset correction identified through visual inspection of dive profiles, and a minimum depth for dives of 2 m, to obtain information about the maximum depth, duration, and descent rate of dives. Dives with unrealistic depths (exceeding depths of the ocean floor around Caamaño, likely due to tag malfunction, representing under 0.01% of dives) were excluded from further analysis. All dives were further analyzed using the automated broken stick algorithm as described by Heerah et al. (2014). In contrast to the traditional division into descent, bottom time and ascent, this approach captures more complex dive profiles by dividing dives in as many segments as necessary to reflect the recorded vertical movement. Individual putative foraging episodes can be determined by analyzing vertical sinuosity of the segments (vertical distance between beginning and end of a segment divided by the sum of all vertical distances in the segment), measuring effectively vertical Area Restricted Search (Heerah et al. 2014). After visually inspecting the histogram of vertical sinuosity for every broken stick segment of every dive, the threshold for foraging was set at a sinuosity index below 0.9. These episodes can be independent of the classical bottom time, which is often used as a proxy for foraging episodes, but prone to over-simplification of diving behavior. This method allows to calculate the duration of foraging, mean depth of foraging, and especially the range of foraging within a dive, giving new and higher resolution data than the traditional approach (see Fig. 1).

GPS positions were decoded using the DAP processor (Wildlife Computers) and erroneous GPS location estimates were excluded using a speed filter of 25 km/h with the R package *trip* (Sumner 2011). Based on this information and visual inspection of dive patterns, we identified individual foraging trips, which are defined as trips on which animals were farther then 50 m away from the coast of Caamaño and which contained at least 30 foraging dives (i.e., dives with segments of a sinuosity index below 0.9), thereby excluding the shortest trips likely not related to foraging. For each foraging trip, the median and interquartile-range of the dive duration, maximum depth, descent rate, foraging depth, foraging duration and foraging range was calculated, as well as the percentage of dives occurring between 18:00 and 06:00 o’clock (night dives), resulting in 13 variables.

### Identifying strategies

To cluster the foraging trips into different foraging strategies, we first checked the correlation matrix of the 13 identified variables and excluded all variables that were highly correlated. The remaining variables were standardized and included in a hierarchical cluster analysis (HCA) using Euclidian distance and Ward’s method. Meaningful clusters were visually identified with the help of a dendrogram (Hennig 2015). Jackknife resampling of foraging trips was used to test for stability of identified clusters (Andes 1989). We used ANOVA and post-hoc Tukey tests to investigate cluster differences in SMI, mass, age, and the variables included in the HCA, and used this information to determine biological differences between the clusters.

### Hidden Markov models

To deepen our understanding of the differences between foraging strategies, hidden Markov models (HMMs) were fitted to individuals while at sea. HMMs can be used to infer unobserved or “hidden” states underlying the observed diving patterns, which may be interpreted as proxies for the behavioral modes of an animal, such as foraging, searching, or traveling (Patterson et al. 2017; McClintock et al. 2020). The state process is modelled as a finite-state Markov chain, with the distribution of each observation completely determined by the underlying state (Zucchini et al. 2016). This approach has been successfully utilized to identify foraging behavior and areas important for conservation management (e.g. van Beest et al. 2019). Three variables connected to foraging were entered into each HMM: the aforementioned dive duration, percentage of acceleration peaks, and mean traveling speed. To calculate acceleration peaks, we first removed the static component from the raw acceleration data using a moving average over two seconds. The overall dynamic body acceleration (ODBA) was then calculated by combining the resulting dynamic accelerations from all three axes (Qasem et al. 2012). To identify ODBA peaks, we manually determined a threshold at the value of six after visual inspection of several ODBA graphs and calculated the percentage of ODBA values above this threshold for each dive. While ODBA is seen as a proxy for the activity of an animal (e.g. Volpov et al. 2015), concentrating on ODBA peaks allows to measure bursts of acceleration, often found during prey chasing attempts. Mean traveling speed of each dive was calculated by dividing a dive’s step length (calculated as the distance between interpolated GPS positions for the start and end points of a dive) with the dive’s duration. Traveling speed thus reflects the relative horizontal expansion of dives, with low traveling speed indicating tortuous movements which are seen as a proxy for foraging (Patterson et al. 2017), also called Area Restricted Search (Dragon et al. 2012). Combining dive duration, horizontal movement, and acceleration data into one model is likely to capture behavioral states more accurately than models that do not use such a multivariate HMM approach (McClintock et al. 2017).

We assume that the observations within a dive are conditionally independent of each other, such that given the states, we can use univariate distributions for each variable. For traveling speed and dive duration, a gamma distribution was chosen, as these variables are positive continuous, while for ODBA peak percentages, a beta distribution with additional point mass on zero was used, as this variable ranges from zero to one and includes dives without any peaks. Those three variables, chosen as indicators of foraging events, were then used to fit separate HMMs to each individual, which allowed us to account for the high variability of individual behavior found in Galápagos sea lions. Individual foraging trips were assumed to be independent of each other. We estimated the model parameters by numerically maximizing the likelihood in R, evaluated with the efficient forward algorithm (cf. Zucchini et al. 2016), using the optimization routine ‘nlm’. To avoid local maxima, we ran each HMM ten times using random starting values and selected the model with the highest likelihood. We fitted two, three and four-state HMMs, which we inspected by plotting their estimated state-dependent distributions and considering their Viterbi-decoded state sequence as well as GPS positions of the decoded states. Finally, we selected the number of states most (biologically) plausible, as suggested by Pohle et al. (2017). Model checking was achieved by graphical comparison of the marginal distributions under the fitted HMM and the empirical distributions, to check the adequacy of the state-dependent distributions. The distribution of the dive parameters within states, as well as the spatial and temporal distribution of decoded states were used to interpret their biological function and assign a behavioral state to them (for more information on the HMM analysis find the R code with an example dataset in the Appendix). To enable comparisons between groups, the states of animals within a foraging strategy were combined in a group summary if the state-dependent distributions of the three variables and the locations of the decoded dive sequences revealed similar patterns and shared the same interpretation of the biological function.

All analyses were performed in R version 4.0.0 (R Core Team; 2020) in the RStudio environment (RStudio Team (2020). RStudio: Integrated Development for R. RStudio, PBC, Boston, MA). Data were mapped using QGIS (QGIS Development Team (2020). QGIS Geographic Information System. Open Source Geospatial Foundation Project).

## Results

### Dive analysis

For this study, we collected an absolute of 595 days of data, comprising 87,109 dives, across all 39 females varying in weight between 51 and 89 kg. The subsequent broken stick analysis identified 51,449 dives with individual segments of a vertical sinuosity index below 0.9, indicating putative foraging dives. GPS locations revealed a total of 177 foraging trips, with 2–9 trips per female and a median duration of 55 h (range: 6–224 h).

### Clustering of foraging trips

**Fig. 2:**
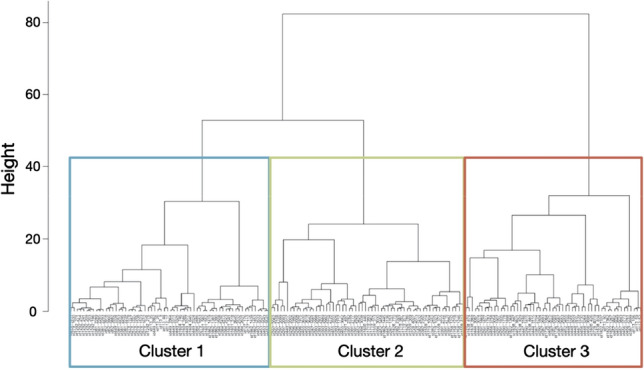
Dendrogram of 177 foraging trips clustered with a hierarchical cluster analysis (Euclidean distance, Ward’s method) into three clusters (cluster 1 = 62 trips, cluster 2 = 60 trips, cluster 3 = 55 trips)

After excluding highly correlated variables from the analysis (correlation coefficient > 0.7), the remaining eight variables used in the hierarchical cluster analysis were median and interquartile range of foraging depth, foraging duration, and range of foraging episodes, the median descent rate, and the percentage of dives at night for each foraging trip. After visual inspection of the resulting dendrogram, three clusters were identified (Fig. 2). Through Jackknive resampling of the trips, a high stability of the three clusters was confirmed (cluster 1 = 89.8%, cluster 2 = 97.7%, cluster 3 = 98.3%).

The three identified clusters are of similar size and contain 62, 60 and 55 foraging trips, respectively. Of the 39 adult females, 31 had all of their foraging trips within one of these clusters while eight animals had foraging trips in two different clusters. Three of those animals had only a single trip (representing 12.5–20% of their trips) in another cluster, which is why we included those animals into their main cluster for later group comparisons. The other five animals were excluded from all following group comparisons, leaving 12, 12 and 10 animals in clusters one, two and three, respectively (see Table 1 in the Appendix).

**Fig. 3:**
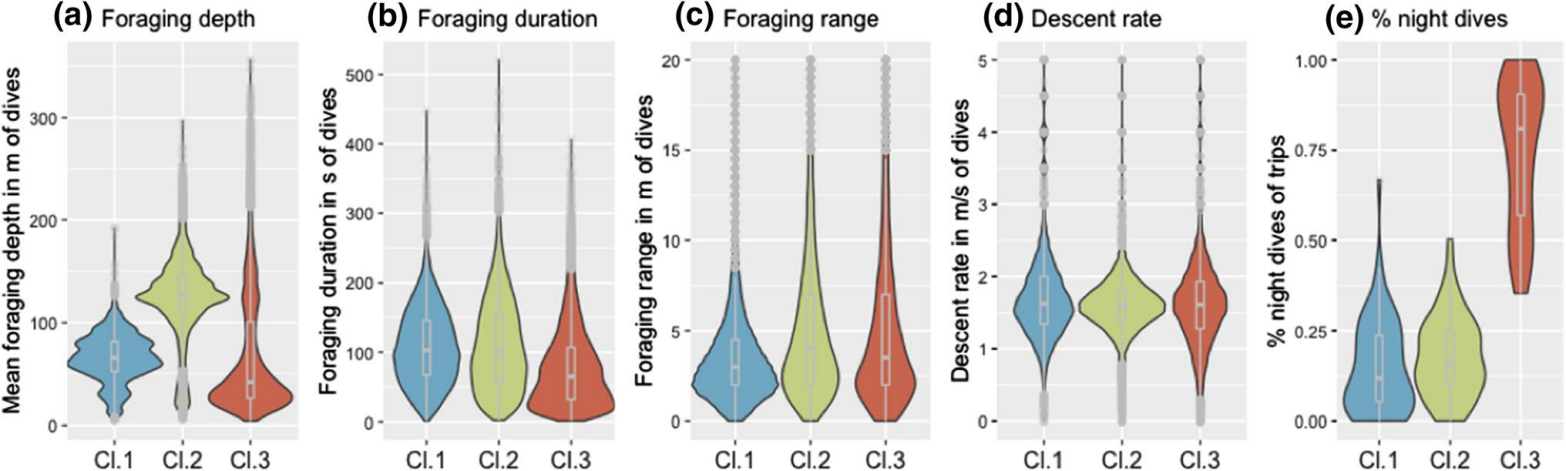
Visual comparison of the dive variables (**a–e**) between the three identified clusters (Cl.1, Cl.2, Cl.3) from the hierarchical cluster analysis through violin plots (cluster 1: *n* = 62, cluster 2: *n* = 60, cluster 3: *n* = 55)

The calculated dive statistics for each cluster reveal clear differences between the strategies. Cluster one has the smallest interquartile range of foraging depth, the smallest foraging range in comparison to the other two clusters, and comprises relatively shallow dives. This pattern would be expected of animals diving along the seabed, thus seeking out similar depths over different dives and showing a small foraging range within a dive. Cluster one is, therefore, defined as **benthic divers**. Cluster two contains the deepest foraging episodes with a high foraging range within dives and a high interquartile-range of foraging depth. This pattern can be interpreted as foraging in the open water column, where the seafloor is not restricting foraging depth. It is, therefore, defined as **pelagic divers**. Cluster three has significantly more dives during the night than the other two clusters, showing high foraging range with a low foraging depth. This cluster is, therefore, defined as **night divers** (Fig. 3). For more detailed differences between the clusters see Table 2 in the Appendix. SMI, mass and age had no explanatory value for assignment to any of the clusters (SMI: F(2,21) = 0.1, p = 0.906; mass: F(2,21) = 0.224, p = 0.801; age: F(2,14) = 0.364, p = 0.701).

### Hidden Markov models

Despite the variation between individuals, similarities of state characteristics within groups are still high while differing from states of other groups. The clearest difference between the three foraging groups was the number of states needed to fit the diving behavior. While most animals of night and pelagic divers are best explained by a three-state model, for benthic divers a two-state model is most adequate with respect to producing biologically meaningful states (in the corresponding three-state models, two states were very similar and could not be reasonably matched to different behaviors). For benthic and pelagic divers, all animals show similar patterns within their respective groups and hence are included in the group summary. Seven of the ten-night divers show similar state characteristics and are used in the group summary, while three-night divers differed clearly from the rest, either in the number of states or the state-dependent distribution, and thus will be discussed separately. The estimated parameters of the state-dependent distributions of all 39 fitted HMMs are found in Table 3 in the Appendix.

**Fig. 4:**
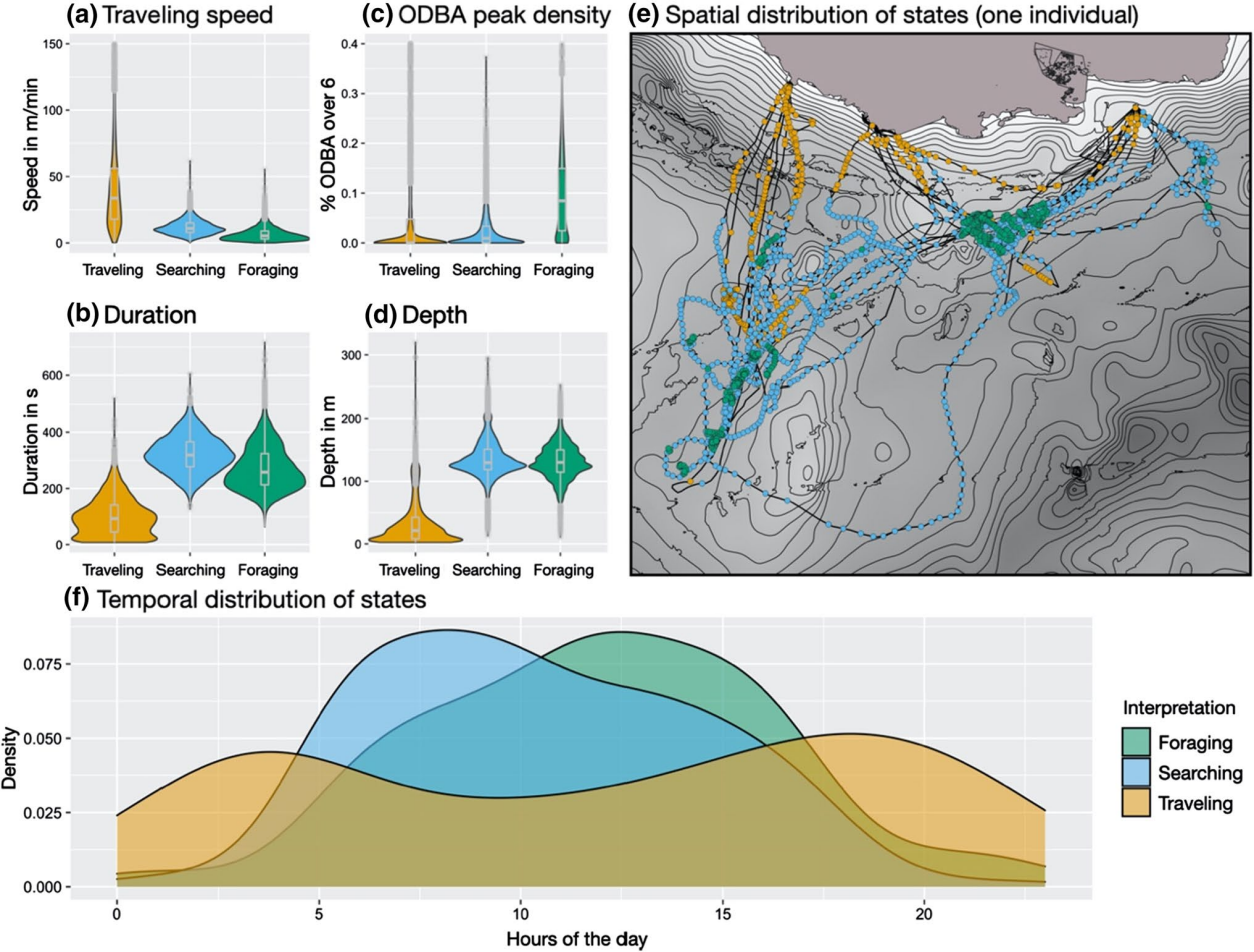
Group summary of pelagic divers’ HMM results, comparing the three states by dive variables (**a**–**d**), their time distribution over 24 h (**f**) (12 animals with 31 foraging trips), as well as an example of the spatial distribution of states for one pelagic diver (**e**) (id1719)

*Pelagic:* State 1 of pelagic divers is characterized by the highest traveling speed, short dive time, and shallow depths. Those short, shallow dives with directed movement are often found immediately after an animal left or returned to a beach (Fig. 4). The temporal distribution also shows that this state is mostly active in the beginning and end of a foraging trip. State 1 is, therefore, interpreted as a traveling state. State 2 differs from the traveling state by deeper and longer dives and an intermediate traveling speed, while ODBA peak percentages are low as within the traveling state, indicating an absence of foraging. Looking at the GPS positions of dives that most likely belong to state 2, one can identify search patterns with wide winding curves covering large areas. State 2 is thus described as a searching state. While similar in depth and dive duration to the searching state, state 3 has the lowest traveling speed and the highest percentage of ODBA peaks and is consequently considered to be a foraging state. This is supported by the GPS data, showing small, dense spatial clustering of dives of state 3. Based on the decoded states, 45% (range: 36–59%) of all dives are classified as putative foraging, while 37% (range: 18–51%) are classified as searching, and 18% (range: 10–42%) as traveling.

**Fig. 5:**
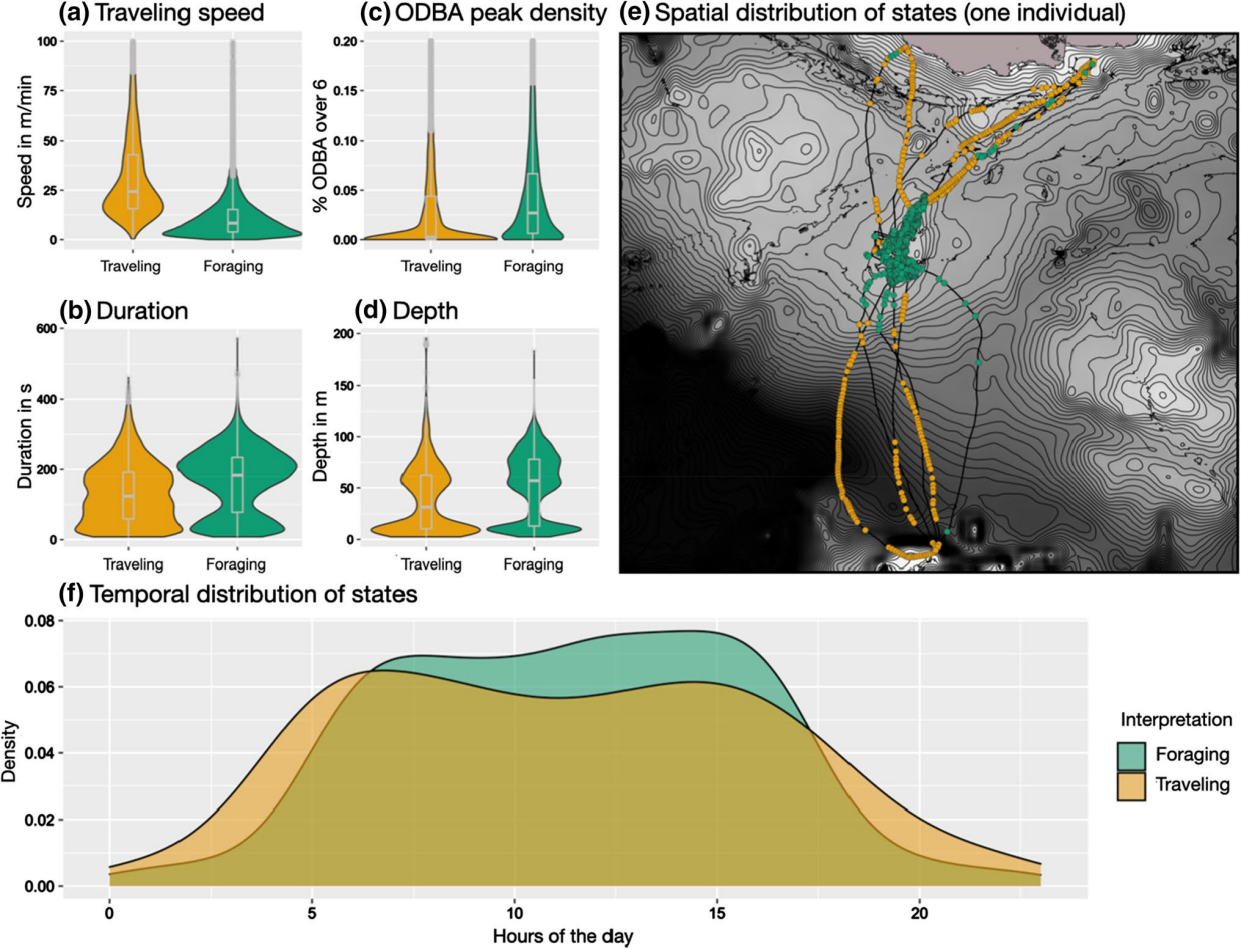
Group summary of benthic divers’ HMM results, comparing the two states by dive variables (**a**–**d**), their time distribution over 24 h (**f**) (12 animals with 57 foraging trips), as well as an example of the spatial distribution of states for one benthic diver (**e**) (id127€)

*Benthic:* State 1 of only two states for benthic divers comprises a high traveling speed and low ODBA peaks, while its spatial and temporal distribution is similar to the traveling state of pelagic divers. Therefore, this state is interpreted as a traveling state (see Fig. 5). State 2 distinguishes itself from the traveling state by lower traveling speed, deeper and longer dives, and a higher percentage of ODBA peaks. State 2 is, therefore, considered to be a foraging state, supported by narrow clustering of the corresponding GPS positions. On average, 26% (range: 12–49%) of dives are classified as traveling and 74% (range: 51–88%) as foraging.

**Fig. 6:**
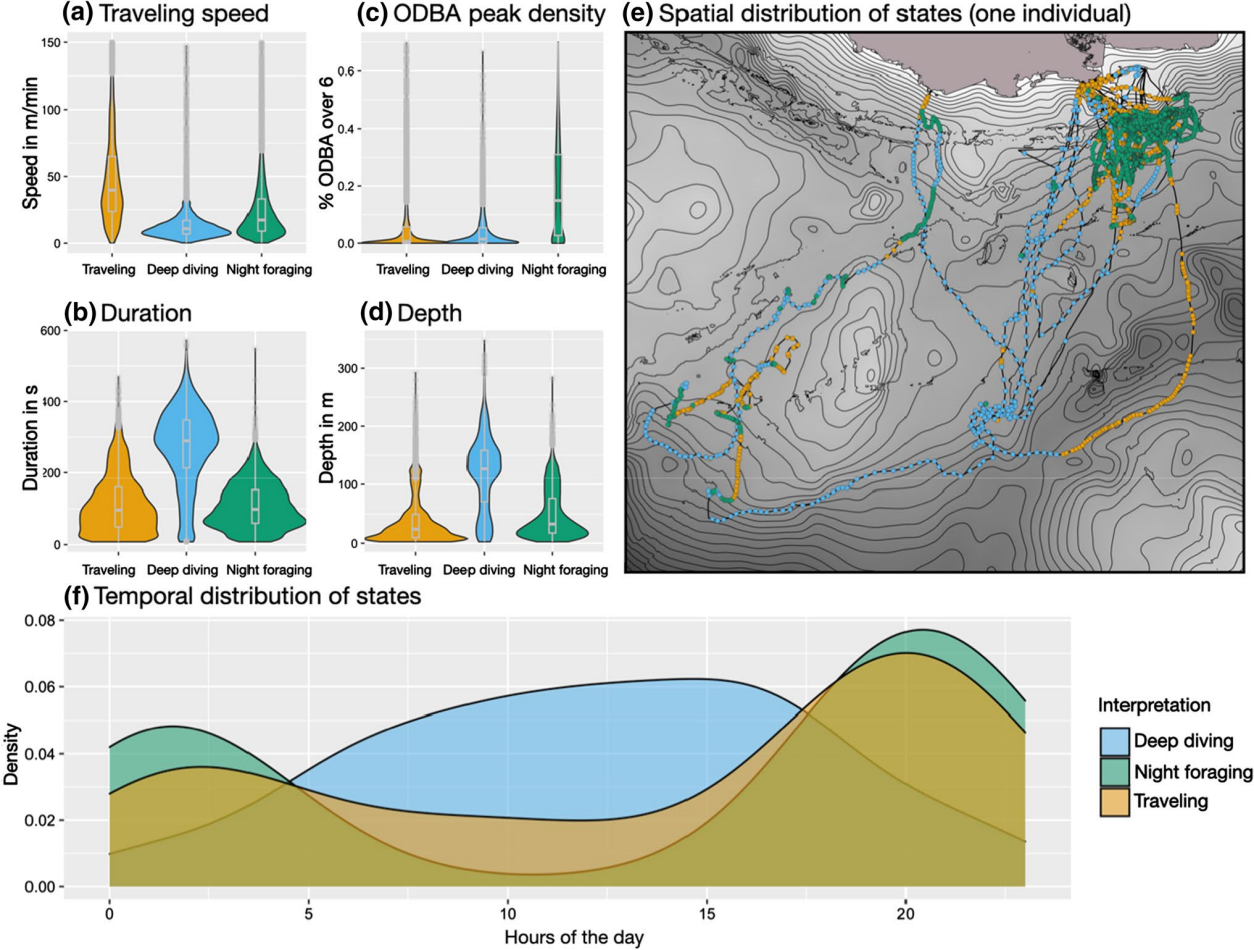
Group summary of night divers’ HMM results, comparing the three states by dive variables (**a**–**d**), their time distribution over 24 h (**f**) (7 animals with 33 foraging trips), as well as an example of the spatial distribution of states for one night diver (**e**) (id1638)

*Night:* State 1 of the night divers can be interpreted as traveling, just like in the two previous groups, due to the high traveling speed, the short and shallow dives and the GPS positions of this state (see Fig. 6). State 2 combines the deepest and longest dives of this group with a low traveling speed, typically indicating foraging, but has still low ODBA peak levels, conflicting with such an interpretation. State 2 is primarily active during the day, while the other two states are primarily found at night. Those daytime deep dives cannot be interpreted further at this stage and are hence called deep dives. State 3 consists of shallow dives with many ODBA bursts and relatively low traveling speed in comparison to the traveling state, indicating foraging. An interpretation of state 3 as night foraging is also supported by the clusters of GPS positions of this state. The three-night divers that were excluded due to their different state patterns all exclusively dove at night. The consequential absence of deep daylight dives explains their different state-dependent distributions compared to the other night divers. Overall, 48% (range: 38–60%) of dives of night divers are classified as night foraging, 20% (range: 11–28%) as deep daylight dives, and 32% (range: 25–43%) as traveling.

*Individuals outside the groups:* Of the five animals that were not included in one of the foraging groups, the diving behavior of four (id447€, id452€, id728, id1600) was best described using a three-state model and showed a high resemblance of their state characteristics to either the night divers or pelagic divers. The remaining animal (id113€) had HMM results that did not resemble the state distributions of any of the three groups.

**Fig. 7:**
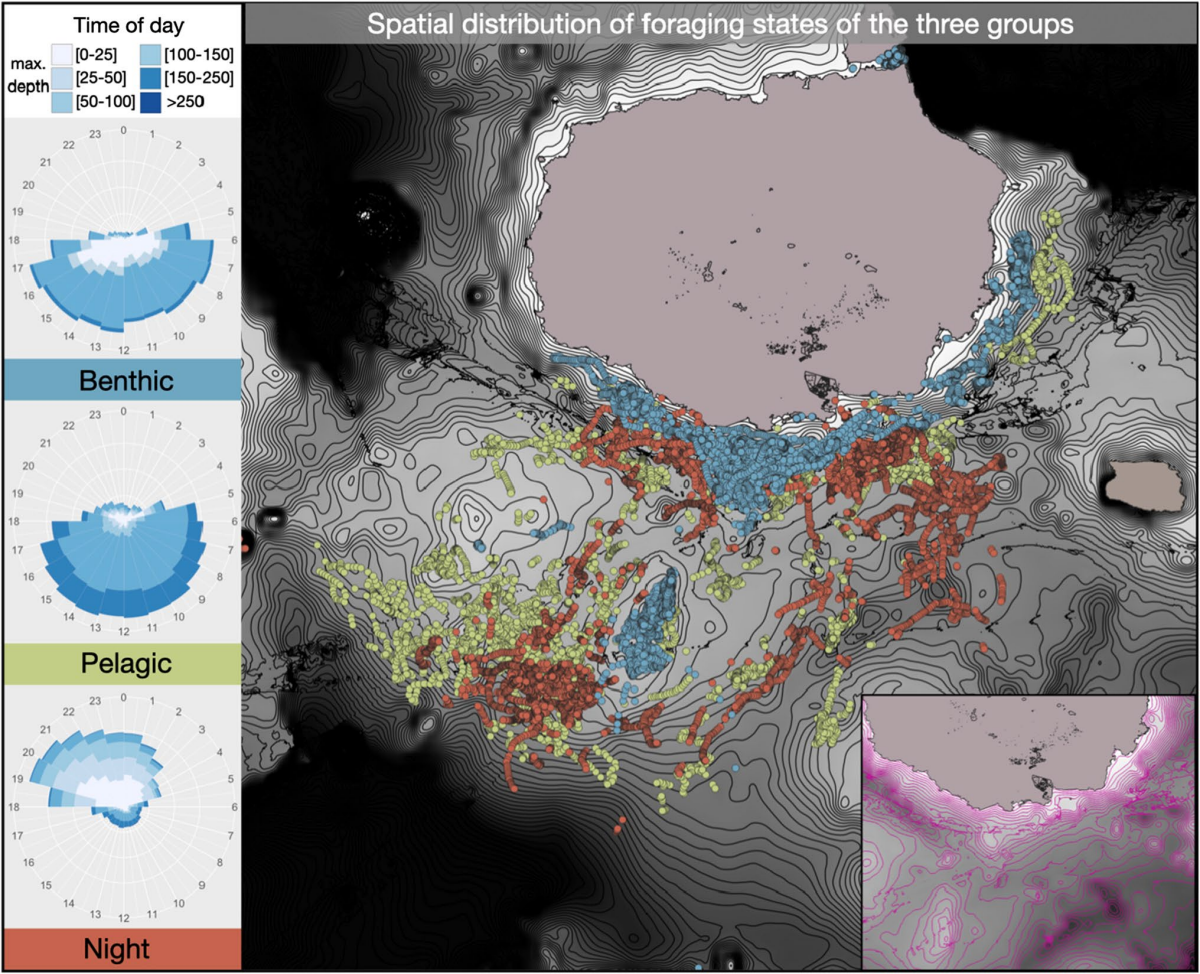
Comparison between benthic, pelagic, and night divers with regard to their distribution of dives with different depths over 24 h (left) and their spatial distribution of dives classified as foraging based on the HHMs (benthic divers (blue): *n* = 12, pelagic divers (green): *n* = 12, night divers (red): *n* = 10) on a bathymetric map (black = 300 m, white = 0 m, lines at 10 m intervals). In the right lower corner, a cutout of the bathymetric map is presented to better visualize shallow areas

*Group comparison:* When comparing the spatial distribution of the groups’ foraging states, the clear spatial demarcation of the benthic divers from the other two groups is striking (see Fig. 7*Spatial distribution*). The foraging dives of benthic divers are concentrated on shallow, flat areas close to the coast of Santa Cruz, on the shallow plateau adjacent to the south, or on top of the underwater mountain found south/west of the island. The foraging areas of the night and pelagic divers hardly overlap with those shallow areas, as their foraging dives are in deeper water, mainly in the underwater valleys between the islands of Santa Cruz in the north and Floreana in the South. The area where foraging dives occur is more extensive for both night and pelagic divers than for benthic divers. While the spatial demarcation between the pelagic and night divers is not distinct, a vertical and temporal separation between those strategies is visible (see Fig. 7*Time of day*). While most dives of night divers occur during the night in shallow depths, pelagic divers show deep foraging dives during the day.

## Discussion

In this work, we leveraged recent advances in telemetry technology and analysis approaches to draw a much more comprehensive picture of the foraging strategies of a marine predator than was previously possible. To our knowledge, we used for the first time a novel set of foraging variables derived from measures of vertical sinuosity within a broken stick algorithm as described by Heerah et al (2014) to identify foraging strategies with a hierarchical cluster analysis in a marine predator. Identifying reliable variables describing foraging is crucial to capture foraging strategies. In previous studies, vertical sinuosity has proven to be a predictor of foraging (e.g. Dragon et al. 2012; Gallon et al. 2013). The combination with the broken stick algorithm facilitates a better identification of episodes with high vertical sinuosity and thus putative foraging events than more traditional approaches such as bottom time (Heerah et al. 2014). Only with these more accurate measures of foraging episodes we were able to calculate some of the new variables describing foraging episodes, such as the depth range of foraging within a dive. This novel set of diving variables allowed for the clear identification of three foraging strategies in the Galápagos sea lions on Caamaño, namely benthic divers, pelagic divers, and night divers. These strategies are to some extent similar to the strategies identified by Villegas-Amtmann et al. (2008) and Villegas-Amtmann and Costa (2010), who studied dive data of 9 and 10 females, respectively, from the Caamaño rookery (deep divers ~ pelagic divers, bottom divers ~ benthic divers, shallow divers ~ night divers). However, these innovative previous studies used the traditional approach of identifying foraging strategies with variables describing dives instead of variables describing foraging within dives by measuring high vertical sinuosity. Hence, the new analytical approach of the current study, as well as the larger sample size and the higher resolution of the dive data, allows us to identify and describe the strategies in more detail, and build on the earlier work.

The additional analysis of individuals’ dive data by multivariate HMMs allowed even more detailed insights into the behavior of individuals when at sea, by approaching the differences between strategies from another angle. To account for the high variability of dive behavior between animals, we decided to fit separate HMMs to the individuals at the cost of a more difficult comparison of the results. This approach is labor-intensive due to the necessary inspection and comparison of the many individual models fitted but had several important advantages over the use of joint models in our setting: (a) mixed HMMs, while able to accommodate heterogeneity, would be restricted in their ability to capture the fundamentally different behavioral strategies we were able to identify, (b) separately fitting joint models to the subgroups identified using cluster analysis would have been viable, but unlike our independent HMM analysis would heavily rely on the adequacy of the clusters identified, and (c) fitting individual models does, in fact, involve a very low computational cost, at least compared to mixed models. Consequently, each individually fitted HMM needs to be analyzed separately to allow for a good pragmatic identification of the number of states which, while suffering a loss in objectivity, is considered to be the best possible practical solution (Pohle et al. 2017). We emphasize that the interpretation of the states from the HMMs should not be taken too literally, since the HMM states do not correspond exactly to the behavioral states and must be interpreted with caution. The following summary of animals with similar state characteristics within groups is a simplification of the HMM analysis but represents a reasonable approach to highlight the main differences between the three main foraging groups.

### Pelagic divers

The interpretation of the three states of the pelagic divers was strongly supported by GPS data and the temporal distribution of states. Travelling dives were often located close to beaches, searching dives covered large areas and foraging dives clustered in deep waters. Considering the temporal distribution, only the observed state sequence of traveling–searching–foraging–traveling appears biologically sensible. These animals search and hunt for food mainly at depths between 100 and 200 m, the epipelagic environment, where they seem to utilize high-density prey patches, as the dense clustering of dives in the foraging state suggests. For sea lions, such prey patches can represent schools of fish or squid, which are typically highly mobile and show low site fidelity, resulting in unpredictable, high-density patches that provide a rich food source if successfully located. This specialization makes a targeted visitation of foraging locations difficult and searching for schools a necessity. The existence of a searching state and the large area covered during foraging trips of pelagic divers, emphasize the specialization of this foraging group on schooling prey.

### Benthic divers

The absence of a searching state differentiates the benthic divers from the pelagic divers and can be an indicator that these animals do not utilize dense prey patches, but rather prey on more solitary living benthic fish. Searching and foraging are combined within one state, with searching being interrupted by short periods of foraging. Benthic fish typically show a higher site fidelity in comparison to schooling pelagic fish and live in benthic communities providing static navigational cues that individuals can use to repeatedly visit the same areas. The benefit of benthic foraging thus lies in a predictable, evenly distributed prey source, albeit occurring at low densities within a habitat (Camprasse et al. 2017). Such predictable foraging habitats can be observed in many of the benthic divers when inspecting their GPS positions of dives, where they target the same specific areas over several foraging trips. The utilized area and habitat differ considerably between individuals but have in common to be rather shallow and flat seabed with foraging dives depths less than 100 m.

### Night divers

Two of the three states of the night divers can clearly be interpreted as traveling and night foraging. Foraging at night happened mostly in very shallow depths of ca. 30 m, reaching occasionally depths of up to 100 m. The observed foraging depths at nightfall fall in line with the vertical migration of mesopelagic fish, such as *Myctophidae*. These deep-sea fish migrate at night from depths between 300 and 1200 m to around 10–100 m to forage on plankton (Nelson 2016), overlapping in their vertical and temporal distribution with the foraging state of the night divers. *Myctophidae* represents a large proportion of prey eaten by Caamaño animals (Páez-Rosas and Aurioles-Gamboa 2014) and are probably targeted specifically by night divers. This strategy resembles the dive behavior of Galápagos fur seals, which also exploit vertically migrating prey during the night (Dellinger and Trillmich 1999). However, interpretation of the states of night divers need extra caution. These animals are not diving exclusively at night but are found to do deep dives during the day. It is reasonable to assume that these animals utilize different strategies during the night and during the day since the prey distribution changes drastically. If we assume two different strategies in the night divers, more than four states might be necessary to find a model that more accurately captures night and day strategies. The fitted three-state model is, therefore, only an approximation, sufficient to highlight the different behaviors of night divers between day and night and the differences toward the other groups.

### Group comparison

These three strategies differed clearly in their dive parameters but not in the animals’ age, body condition, or mass. This implies that body condition (SMI) of these sea lions is not a factor driving different strategies in this species nor do differences in experience (age) or physiological limitations, like oxygen store capability, due to size (mass), as found in other pinniped species (Costa et al. 2004; Weise and Costa 2007). The missing influence of mass on an animal’s foraging strategy could be due to the relative shallow waters utilized by sea lions of the central part of the archipelago, which rarely exceed 300 m. One-year-old juveniles (below 30 kg) were shown to dive already below 350 m (Jeglinski et al 2012), and maximum depths of 584 m recorded for adult female Galápagos sea lions from different colonies demonstrate that, in the shallow waters around Caamaño, size is not the limiting factor for the dive behavior (Jeglinski et al. 2013). These findings indicate that individual foraging strategies of centrally located Galápagos sea lion females might be shaped by behavior or genes than by age or mass characteristics (Chilvers and Wilkinson 2009).

Our novel combination of analytic approaches did not only identify differences between animals of different foraging strategies in dive parameters, but also in their preferences of habitats, composition of identified states, and their temporal distribution. This highlights the importance of such a multivariate approach to understand the different facets of foraging. These differences are so pronounced that there is little overlap between animals of different groups, either because of a clear spatial separation of foraging habitats (benthic versus others), or because of a temporal and vertical separation (pelagic versus night). This separation might reduce intraspecific competition, as described in Antarctic fur seals (Kernaléguen et al. 2015) and discussed for Galápagos sea lions (Páez-Rosas et al. 2017), representing a strong driver for the development and stabilization of those foraging strategies. Avoidance of competition, however, might not be the only driver for the observed individual differences. Other possible drivers for foraging strategy diversity might be adaptiveness toward different environmental conditions or early experiences during the learning phase of foraging.

When discussing assumptions about the adaptive value of foraging strategies toward different environments and possible fitness consequences, knowledge of the stability of strategies over a long time period within individuals is needed. In this study, the stability of foraging strategies was shown over two to three weeks. Though this study can only infer the stability of foraging strategies over deployment time, studies of other sea lion species showed a stability of individual foraging strategies over several seasons (Lowther et al. 2011, 2013; McHuron et al. 2018), suggesting that a similar stability could exist in Galápagos sea lions. This does not mean that sea lions cannot alter their response to changing environments, but it does mean that despite behavioral changes, a strong persistence of foraging strategies might prevail. Stability might allow a better specialization toward one foraging strategy. This may not only include increasing knowledge of the spatial distribution of prey but may also entail optimization of specialized foraging techniques. This could be expected to vary especially in foraging strategies that differ as fundamentally as found in this study, such as between benthic divers and the two other strategies. Although stability of individual foraging strategies has to be studied further in Galápagos sea lions, with the current data we can cautiously assume the stability of individual strategies.

Adaptive value of individual strategies under different conditions is an important topic when studying foraging strategies since they allow us to infer resilience in the face of future environmental change. In our study, the different specializations of strategies are consistent with prey species found in the diet of the Galápagos sea lions (Páez-Rosas and Aurioles-Gamboa 2014). Benthic fish, which are mainly targeted by benthic divers, often have lower lipid content (Anthony et al. 2000), and their higher distribution can result in less efficient foraging compared to hunting pelagic schooling fish (Costa and Gales 2003; Chilvers and Wilkinson 2009). Benthic divers might, therefore, face a disadvantage compared to strategies specializing on pelagic fish if the environment is stable. However, the Galápagos islands are exposed to annual changes in sea surface temperature and to unpredictable weather events such as El Niños. The resultant exceptionally high sea surface temperatures with low marine primary productivity causes severe shifts in prey availability (Feldman et al 1984; Trillmich and Limberger 1985). Fish species with low site fidelity, such as most pelagic fish, often leave to seek other feeding grounds (Arntz et al. 1991), while fish species with higher site fidelity, such as many benthic fish species, are less able to (Miller and Sydeman 2004). Under such conditions of higher sea surface temperature during El Niño events, benthic divers might have an advantage over animals specialized on pelagic fish species. Benthic divers might, therefore, follow a risk-averse foraging strategy, targeting reliable but possibly low-yield environments. With climate change, El Niño events are expected to occur more regularly and to become more extreme (Trenberth and Hoar 1997; but see Cobb et al. 2013). Knowledge of the coping capability of different strategies might allow us to model population dynamics in more detail, producing information needed for the management of this endangered species in addition to traditional methods, such as the establishment of protected areas based on the identified foraging habitat (Ventura et al. 2019).

On the whole, we could demonstrate the advantages of our new combination of analysis approaches over traditional approaches to identify and describe foraging strategies in a marine predator in great detail. These new insights into the behavior of Galápagos sea lions at sea may help derive possible adaptive consequences of these strategies. Our results represent a milestone in the study of foraging strategies of Galápagos sea lions and their ecological niche. We also demonstrate the importance of a multivariate approach using high-resolution data to identify foraging strategies in predators and to address individual differences to better describe and understand their ecological diversity.

## Supplementary Information

Below is the link to the electronic supplementary material.

Supplementary file1 (DOCX 153 KB)

Supplementary file2 (RTF 13 KB)

Supplementary file3 (CSV 212 KB)

## Data Availability

The dataset used during the current study is available from the corresponding author on reasonable request. A sample dataset and code are provided in the Electronic Supplementary Material.
