# Supplementary material for: Individuality counts: A new comprehensive approach to foraging strategies of a tropical marine predator
Source: Oecologia. 2021 Jan 24;195(2):313–25. doi: 10.1007/s00442-021-04850-w (PMC7882564; doi:10.1007/s00442-021-04850-w)
Supplement: Supplementary file 1 — Supplementary file1 (DOCX 153 KB) [file 442_2021_4850_MOESM1_ESM.docx]

Electronic Supplementary Material to:

Individuality counts: A new comprehensive approach to foraging strategies of a tropical marine predator

Jonas F.L. Schwarz^1*^, Sina Mews^2^, Eugene J. DeRango^1^, Roland Langrock^2^, Paolo Piedrahita^3^, Diego Páez-Rosas^4,5^, Oliver Krüger^1^

^1^ Department of Animal Behaviour, Bielefeld University, Bielefeld, Germany

^2^ Department of Business Administration and Economics, Bielefeld University, Bielefeld, Germany

^3^ Facultad de Ciencias de la Vida, Escuela Superior Politécnica del Litoral, Guayaquil, Ecuador

^4^ Universidad San Francisco de Quito, Galápagos Science Center, Isla San Cristobal, Galápagos, Ecuador

^5^ Dirección Parque Nacional Galápagos, Unidad Técnica Operativa San Cristóbal, Isla San Cristobal, Galápagos, Ecuador

*Correspondence: jonas.fl.schwarz@gmail.com

**Table 1** Overview of all females (N=39) and their distribution of trips into the three foraging groups based on the hierarchical cluster analysis, as well as their age, scaled mass index (SMI), and duration of TDR deployment. Animals marked in red were excluded from group comparisons

| Id | Benthic trips | Pelagic trips | Night trips | Age  (years) | SMI | Deployment (days) |
| --- | --- | --- | --- | --- | --- | --- |
| 1292 | 7 | - | - | 11 | 58 | 22 |
| 278€ | 6 | - | - | - | 69 | 9 |
| 279€ | 3 | - | - | - | 74 | 7 |
| 453€ | 4 | - | - | - | 76 | 21 |
| 127€ | 2 | - | - | 15 | 71 | 13 |
| 1604 | 3 | - | - | 12 | 68 | 13 |
| 381€ | 6 | - | - | - | 68 | 18 |
| 384€ | 7 | - | - | 13 | 81 | 13 |
| 52€ | 3 | - | - | 18 | 72 | 7 |
| 449€ | 7 | 1 | - | 9 | 80 | 19 |
| 1862 | 5 | 1 | - | 8 | 78 | 14 |
| 1601 | 4 | 1 | - | 12 | 65 | 15 |
| 113€ | 5 | - | 4 | 13 | - | 15 |
| 1559 | - | 5 | - | 9 | 73 | 20 |
| 1758 | - | 3 | - | 8 | 69 | 17 |
| 297€ | - | 5 | - | 14 | 79 | 12 |
| 455€ | - | 5 | - | - | 87 | 14 |
| 1626 | - | 2 | - | 15 | 65 | 3 |
| 1719 | - | 6 | - | 7 | 68 | 22 |
| 287€ | - | 3 | - | - | - | 12 |
| 296€ | - | 4 | - | 16 | 72 | 16 |
| 382€ | - | 4 | - | 14 | 66 | 15 |
| 391€ | - | 7 | - | 16 | 76 | 21 |
| 393€ | - | 5 | - | 14 | 71 | 10 |
| 394€ | - | 2 | - | 16 | 66 | 10 |
| 447€ | - | 1 | 2 | 13 | 59 | 16 |
| 1600 | - | 1 | 1 | 17 | 55 | 10 |
| 452€ | - | 2 | 1 | 16 | 65 | 14 |
| 728 | - | 2 | 1 | 14 | 73 | 14 |
| 1822 | - | - | 4 | 7 | 61 | 15 |
| 290€ | - | - | 4 | 16 | 86 | 18 |
| 291€ | - | - | 3 | - | 76 | 21 |
| 444€ | - | - | 4 | 13 | 77 | 18 |
| 448€ | - | - | 4 | 14 | 68 | 11 |
| 456€ | - | - | 6 | 11 | 80 | 17 |
| 457€ | - | - | 4 | - | 69 | 14 |
| 1638 | - | - | 6 | 16 | - | 16 |
| 1736 | - | - | 3 | 7 | 63 | 11 |
| 387€ | - | - | 6 | 10 | 67 | 20 |

**Table 2** Statistical comparison of the variables analyzed by the broken stick algorithm and the percentage of dives occurring at night during foraging trips between the three identified clusters from the hierarchical cluster analysis (cluster1 n=62, cluster2 n=60, cluster3 n=55)

**

**Table 3** State-dependent distributions of HMMs fitted to the 39 individuals

|  | *Dive duration (mu/sigma)* | | | *Traveling speed (mu/sigma)* | | | *ODBA peak density (mu/sigma/nu)* | | |
| --- | --- | --- | --- | --- | --- | --- | --- | --- | --- |
| *Animals* | *State 1* | *State 2* | *State 3* | *State 1* | *State 2* | *State 3* | *State 1* | *State 2* | *State 3* |
| 1292 | 166/71.3 | 177/43.1 | - | 57.4/16.8 | 24.7/16.4 | - | 0.08/0.25/0.41 | 0.1/0.24/0.09 | - |
| 278€ | 250/77.0 | 254/35.2 | - | 52.9/20.2 | 16.8/13.6 | - | 0.03/0.13/0.15 | 0.04/0.10/0.01 | - |
| 279€ | 105/63.5 | 201/46.3 | - | 54.5/32.4 | 39.6/24.5 | - | 0.13/0.35/0.92 | 0.03/0.14/0.23 | - |
| 453€ | 164/102.1 | 200/39.2 | - | 75.5/26.3 | 27.7/20.3 | - | 0.06/0.27/3.15 | 0.05/0.20/0.52 | - |
| 127€ | 105/63.0 | 224/36.3 | - | 87.0/28.1 | 31.2/19.9 | - | 0.07/0.35/11.3 | 0.03/0.13/0.1 | - |
| 1604 | 65.1/41.3 | 38.2/22.6 | - | 66.0/31.6 | 14.1/11.3 | - | 0.10/0.32/0.39 | 0.37/0.49/0.12 | - |
| 381€ | 86.5/64.8 | 201.1/62.6 | - | 60.1/26.1 | 44.5/22.3 | - | 0.11/0.29/1.35 | 0.07/0.23/0.15 | - |
| 384€ | 167/134.8 | 283/43.1 | - | 76.8/25.1 | 20.6/15.0 | - | 0.04/0.21/3.00 | 0.02/0.09/0.06 | - |
| 52€ | 241/69.1 | 147/72.1 | - | 50.1/13.9 | 31.2/21.5 | - | 0.03/0.14/1.35 | 0.07/0.19/0.65 | - |
| 449€ | 59.1/44.9 | 53.4/27.3 | - | 68.32/29.7 | 9.83/6.97 | - | 0.15/0.42/2.69 | 0.24/0.45/0.28 | - |
| 1862 | 191/100 | 214/37.7 | - | 57.5/32.1 | 15.2/11.4 | - | 0.06/0.27/0.37 | 0.06/0.21/0.06 | - |
| 1601 | 218/100.8 | 262/27.6 | - | 65.6/39.68 | 13.0/8.69 | - | 0.06/0.30/1.11 | 0.01/0.05/0.36 | - |
| 113€ | 125/67.0 | 102/39.5 | 154/25.9 | 71.7/22.75 | 34.9/23.65 | 10.4/8.41 | 0.05/0.20/0.64 | 0.16/0.33/0.18 | 0.03/0.12/0.11 |
| 1559 | 71.7/53.2 | 290.1/53.8 | 224.5/42.5 | 53.4/39.4 | 63.3/31.7 | 38.9/22.6 | 0.27/0.51/0.61 | 0.06/0.22/0.03 | 0.13/0.32/0.10 |
| 1758 | 97.2/65.8 | 265.3/53.9 | 216.9/51.2 | 61.8/32.1 | 68.2/23.2 | 31.0/17.7 | 0.23/0.53/0.99 | 0.04/0.25/0.42 | 0.04/0.32/0.12 |
| 297€ | 148/99.2 | 372/49.7 | 345/49.3 | 46.1/21.2 | 55.3/22.6 | 19.5/14.1 | 0.12/0.38/0.52 | 0.05/0.20/0.21 | 0.11/0.20/0.01 |
| 455€ | 122/115.0 | 391/41.0 | 339/77.9 | 68.1/36.8 | 71.0/16.4 | 42.0/21.6 | 0.10/0.38/2.15 | 0.01/0.08/0.31 | 0.05/0.23/0.12 |
| 1626 | 210/86.6 | 308/36.3 | 281/31.5 | 49.8/37.2 | 74.3/19.1 | 29.7/14.3 | 0.10/0.29/0.63 | 0.02/0.12/0.46 | 0.05/0.18/0.09 |
| 1719 | 116/68.1 | 281/48.1 | 210/36.3 | 72.1/40.3 | 60.6/23.7 | 20.0/13.8 | 0.11/0.32/0.97 | 0.03/0.20/0.43 | 0.14/0.23/0.14 |
| 287€ | 114/74.1 | 354/52.1 | 308/68.8 | 87.3/21.7 | 62.8/20.6 | 23.1/15.8 | 0.28/0.69/8.93 | 0.03/0.15/0.20 | 0.09/0.24/0.07 |
| 296€ | 99.9/93.5 | 368.0/63.0 | 370.2/75.6 | 47.6/33.6 | 48.7/19.2 | 19.9/11.6 | 0.24/0.49/1.12 | 0.04/0.19/0.22 | 0.07/0.24/0.10 |
| 382€ | 118/90.5 | 290/44.2 | 221/39.7 | 68.5/31.4 | 66.7/22.3 | 22.7/16.4 | 0.05/0.25/3.73 | 0.03/0.20/0.67 | 0.12/0.26/0.25 |
| 391€ | 164/125.3 | 339/55.9 | 257/48.9 | 56.0/28.8 | 52.7/18.3 | 19.9/12.9 | 0.07/0.34/8.06 | 0.01/0.08/0.94 | 0.10/0.23/0.12 |
| 393€ | 123/78.2 | 439/44.3 | 361/52.4 | 80.2/28.5 | 74.9/15.8 | 28.2/17.1 | 0.09/0.42/4.69 | 0.03/0.19/0.05 | 0.06/0.18/0.06 |
| 394€ | 104/90.3 | 338/31.7 | 271/42.5 | 53.0/39.7 | 46.6/24.7 | 32.9/19.9 | 0.24/0.53/1.53 | 0.04/0.18/0.05 | 0.12/0.29/0.11 |
| 447€ | 79.9 /64.0 | 299.2/45.0 | 112.8/86.9 | 65.2/21.8 | 26.4/23.1 | 54.4/13.4 | 0.18/0.38/0.43 | 0.03/0.18/0.12 | 0.28/0.43/0.03 |
| 1600 | 74.7/63.4 | 368.9 /55.6 | 200.0/121 | 72.5/14.6 | 32.2/18.4 | 38.0/18.7 | 0.11/0.36/0.95 | 0.03/0.14/0.06 | 0.14/0.43/0.19 |
| 452€ | 77.9/56.5 | 190.3/154 | 290.5/50.5 | 42.4/22.4 | 93.6/16.8 | 30.2/22.3 | 0.25/0.49/0.79 | 0.04/0.24/1.50 | 0.06/0.23/0.06 |
| 728 | 153/85.4 | 336/46.9 | 264/54.3 | 52.9/43.1 | 58.2/19.1 | 27.8/18.2 | 0.17/0.49/0.76 | 0.02/0.13/0.25 | 0.12/0.32/0.31 |
| 1822 | 86.7/61.8 | 133.8/27.2 | 122.5/29.6 | 62.1/45.7 | 25.3/24.9 | 36.8/25.3 | 0.16/0.40/0.52 | 0.06/0.16/0.02 | 0.29/0.24/0.01 |
| 290€ | 102/80.8 | 325/65.8 | 125/73.3 | 73.6/23.6 | 49.8/27.0 | 28.0/15.1 | 0.14/0.44/1.33 | 0.05/0.24/0.35 | 0.22/0.39/0.21 |
| 291€ | 92.5/82.0 | 295.2/74.7 | 121.0/69.4 | 64.5/22.9 | 56.1/32.2 | 21.6/12.4 | 0.17/0.44/1.67 | 0.06/0.27/0.54 | 0.24/0.43/0.15 |
| 444€ | 259/53.4 | 154/124.4 | 109/70.5 | 42.1/24.0 | 82.0/25.6 | 27.0/15.8 | 0.07/0.27/0.28 | 0.13/0.43/1.00 | 0.27/0.41/0.09 |
| 448€ | 103/65.6 | 296/36.9 | 107/50.7 | 71.5/22.9 | 48.0/26.4 | 29.3/13.3 | 0.10/0.38/0.62 | 0.03/0.16/0.09 | 0.19/0.40/0.11 |
| 456€ | 92.8/62.3 | 233.3/191 | 70.2/41.3 | 74.2/19.3 | 46.9/39.0 | 34.5/11.4 | 0.17/0.46/0.53 | 0.10/0.34/0.27 | 0.24/0.48/0.29 |
| 457€ | 145/69.3 | 132/56.0 | - | 49.9/15.3 | 18.2/11.4 | - | 0.13/0.37/0.32 | 0.19/0.39/0.16 | - |
| 1638 | 88.8/72.2 | 347.5/56.9 | 137.2/82.8 | 71.9/25.1 | 63.0/20.5 | 26.2/13.2 | 0.16/0.44/1.26 | 0.03/0.18/0.21 | 0.24/0.40/0.35 |
| 1736 | 87.1/80.1 | 70.3/53.5 | - | 81.5/22.4 | 36.6/18.0 | - | 0.16/0.43/0.51 | 0.29/0.46/0.23 | - |
| 387€ | 105.8/64.3 | 284.8/186 | 44.7/84.9 | 99.8/21.0 | 49.6/21.8 | 48.0/24.8 | 0.07/0.28/1.07 | 0.07/0.30/0.15 | 0.21/0.43/0.17 |
