# Supplementary material for: Individuality counts: A new comprehensive approach to foraging strategies of a tropical marine predator
Source: Oecologia. 2021 Jan 24;195(2):313–25. doi: 10.1007/s00442-021-04850-w (PMC7882564; doi:10.1007/s00442-021-04850-w)
Supplement: Supplementary file 2 — Supplementary file2 (RTF 13 KB) [file 442_2021_4850_MOESM2_ESM.rtf]

##### R code for the HMM analysis of sea lion dive data## Schwarz et al. Individuality counts: A new comprehensive approach to foraging strategies of a tropical marine predator## the code was tested in R (version 4.0.2) on Windows 10 with packages...# ...gamlss (version 5.2.0)# ...stringr (version 1.4.0)# ...rworldmap (version 1.3.6)#### 1. Load the necessary packages library(gamlss)library(stringr)library(rworldmap)#### 2. Reading in the observations of the example female id1719, a pelagic diverobs <- read.csv("example_animal.csv")## ID = ID of female; Trip = Foraging trip ID of dive; Time = Time in hours (!GMT!);## divetime = Duration of dive in secondsobs$divetime <- as.numeric(obs$divetime)## step = Mean traveling speed (step length / dive duration)obs$step <- as.numeric(obs$step)## ODBA = % ODBA peaks above the threshold of 6obs$ODBA <- as.numeric(obs$ODBA)## Latitude = Latitude cooridinates of dive; Longitude = Longitude cooridinates of dive; ## maxdepth = Maximun depth of dive in meters#### 3. Defining the necessary functions:## Function that converts 'natural' parameters (possibly constrained) to 'working' parameters (all of which are real-valued) - ## This is  necessary since we use the unconstrained optimizer nlm() below pn2pw <- function(mu.step, mu.diveDur, mu.odba, sigma.step, sigma.diveDur, sigma.odba, nu.odba, delta, gamma, N){  tmu.step <- log(mu.step)  tmu.diveDur <- log(mu.diveDur)  tmu.odba <- qlogis(mu.odba)  tsigma.step <- log(sigma.step)  tsigma.diveDur <- log(sigma.diveDur)  tsigma.odba <- qlogis(sigma.odba)  tnu.odba <- log(nu.odba)  tdelta <- log(delta[-1]/delta[1])  tgamma <- NULL  foo <- log(gamma/diag(gamma))  tgamma <- as.vector(foo[!diag(N)])  parvect <- c(tmu.step, tmu.diveDur, tmu.odba, tsigma.step, tsigma.diveDur, tsigma.odba,                tnu.odba, tdelta, tgamma)  return(parvect)}## Function that performs the inverse transformationpw2pn <- function(parvect,N){  mu.step <- exp(parvect[1:N])  mu.diveDur <- exp(parvect[(N+1):(2*N)])  mu.odba <- plogis(parvect[(2*N+1):(3*N)])  sigma.step <- exp(parvect[(3*N+1):(4*N)])  sigma.diveDur <- exp(parvect[(4*N+1):(5*N)])  sigma.odba <- plogis(parvect[(5*N+1):(6*N)])  nu.odba <- exp(parvect[(6*N+1):(7*N)])  delta <- c(1, exp(parvect[7*N+1:(N-1)]))  delta <- delta/sum(delta)  gamma <- diag(N)  gamma[!gamma] <- exp(parvect[(8*N):length(parvect)])  gamma <- gamma/apply(gamma,1,sum)  return(list(mu.step=mu.step, mu.diveDur=mu.diveDur, mu.odba=mu.odba,               sigma.step=sigma.step, sigma.diveDur=sigma.diveDur, sigma.odba=sigma.odba,              nu.odba=nu.odba, gamma=gamma, delta=delta))}## Function that computes minus the log-likelihood of the movement HMMmllk <- function(parvect,obs,N){  lpn <- pw2pn(parvect,N)  l.all <- 0  for (k in unique(obs$Trip)) {    obs_trip <- obs[which(obs$Trip==k),]    n <- dim(obs_trip)[1]    allprobs <- matrix(rep(1,N*n), nrow=n)    ind.step <- which(!is.na(obs_trip$step))    for (j in 1:N){      step.prob <- rep(1,n)      step.prob[ind.step] <- dgamma(obs_trip$step[ind.step], shape=lpn$mu.step[j]^2/lpn$sigma.step[j]^2, scale=lpn$sigma.step[j]^2/lpn$mu.step[j])      diveDur.prob <- dgamma(obs_trip$divetime, shape=lpn$mu.diveDur[j]^2/lpn$sigma.diveDur[j]^2, scale=lpn$sigma.diveDur[j]^2/lpn$mu.diveDur[j])      odba.prob <- dBEINF0(obs_trip$ODBA, mu=lpn$mu.odba[j], sigma=lpn$sigma.odba[j], nu=lpn$nu.odba[j])      allprobs[,j] <- step.prob * diveDur.prob * odba.prob    }    foo <- lpn$delta*allprobs[1,]    sumfoo <- sum(foo)    lscale <- log(sumfoo)    foo <- foo/sumfoo    if(n>1){      for (i in 2:n){        foo <- foo%*%lpn$gamma*allprobs[i,]        sumfoo <- sum(foo); lscale <- lscale+log(sumfoo); foo <- foo/sumfoo # scaling      }    }    l.all <- l.all+lscale  }  return(-l.all)}## Function that runs the numerical minimization of 'mllk' (i.e. tries to find the MLE)mle <- function(obs, mu0.step, mu0.diveDur, mu0.odba, sigma0.step, sigma0.diveDur, sigma0.odba, nu0.odba, delta0, gamma0, N){  parvect <- pn2pw(mu0.step, mu0.diveDur, mu0.odba, sigma0.step, sigma0.diveDur, sigma0.odba, nu0.odba, delta0, gamma0, N)  mod <- nlm(mllk, parvect, obs, N, print.level=1, iterlim=1000, stepmax=5)  pn <- pw2pn(mod$estimate, N)  return(list(mu.step=pn$mu.step, mu.diveDur=pn$mu.diveDur, mu.odba=pn$mu.odba,              sigma.step=pn$sigma.step, sigma.diveDur=pn$sigma.diveDur, sigma.odba=pn$sigma.odba,              nu.odba=pn$nu.odba, gamma=pn$gamma, delta=pn$delta, mllk=mod$minimum))}## Viterbi algorithm for state decodingviterbi <- function(mod,obs,N){  states <- vector()  for (k in unique(obs$Trip)) {    obs_trip <- obs[which(obs$Trip==k),]    n <- nrow(obs_trip)    allprobs <- matrix(1,nrow=n,ncol=N)    ind.step <- which(!is.na(obs_trip$step))    for (j in 1:N){      step.prob <- rep(1,n)      step.prob[ind.step] <- dgamma(obs_trip$step[ind.step], shape=mod$mu.step[j]^2/mod$sigma.step[j]^2, scale=mod$sigma.step[j]^2/mod$mu.step[j])      diveDur.prob <- dgamma(obs_trip$divetime, shape=mod$mu.diveDur[j]^2/mod$sigma.diveDur[j]^2, scale=mod$sigma.diveDur[j]^2/mod$mu.diveDur[j])      odba.prob <- dBEINF0(obs_trip$ODBA, mu=mod$mu.odba[j], sigma=mod$sigma.odba[j], nu=mod$nu.odba[j])      allprobs[,j] <- step.prob * diveDur.prob * odba.prob    }    xi <- matrix(0,nrow=n,ncol=N)    foo <- mod$delta*allprobs[1,]    xi[1,] <- foo/sum(foo)    for (t in 2:n){      foo <- apply(xi[t-1,]*mod$gamma,2,max)*allprobs[t,]      xi[t,] <- foo/sum(foo)    }    iv <- numeric(n)    iv[n] <- which.max(xi[n,])    for (t in (n-1):1) {      iv[t] <- which.max(mod$gamma[,iv[t+1]]*xi[t,])    }    states <- c(states, iv)  }  obs$state <- states  return(obs)}#### 4. Exploring the histograms of observations and summary statistics to choose a suitable range for the starting valuespar(mfrow=c(3,1))hist(obs$step,breaks=50,col="darkgray",border="white",xlab="",main="step length")hist(obs$divetime,breaks=50,col="darkgray",border="white",xlab="",main="dive duration")hist(obs$ODBA,breaks=50,col="darkgray",border="white",xlab="",main="ODBA")summary(obs$step)summary(obs$divetime)summary(obs$ODBA)#### 5. Define number of states and number of models to fit N <- 3 # number of states to be fitted (for example 2-4)n.fits <- 10 # number of models to be fitted with random starting valuesmods <- list()llks <- rep(NA, n.fits)#### 6. Fit model with random starting values # WARNING: depending on the specifications of N and n.fits, this code will run for several minutes or even hoursset.seed(135)for (i in 1:n.fits) {  # specify appropriate range for random starting values (shouldn't be too narrow, but shouldn't range from min to max value either)  mu0.step <- runif(N, min=10, max=90)#  sigma0.step <- runif(N, min=5, max=30)  mu0.diveDur <- runif(N, min=100, max=320)#  sigma0.diveDur <- runif(N, min=10, max=120)  mu0.odba <- runif(N, min=0, max=0.15)#  sigma0.odba <- runif(N, min=0, max=0.2)  nu0.odba <- runif(N, min=0.001, max=1.5)  # from here on, no changes needed  delta0 <- runif(N, min=0, max=1)  delta0 <- delta0/sum(delta0)  gamma0 <- diag(N)  gamma0[!gamma0] <- runif(N*(N-1), min=0, max=0.3)  gamma0 <- gamma0/apply(gamma0,1,sum)  mods[[i]] <- mle(obs, mu0.step, mu0.diveDur, mu0.odba, sigma0.step, sigma0.diveDur, sigma0.odba, nu0.odba, delta0, gamma0, N)  llks[i] <- -mods[[i]]$mllk}#### 7. Select model with higest likelihood (higher llks = better model fit)par(mfrow=c(1,1))plot(llks, pch=16, xlab="Index")best <- which.max(llks) # find model with highest likelihood-mods[[best]]$mllkmods[[best]]$states <- viterbi(mods[[best]], obs, N)#### 8. Plot the state-dependent distributions to explore model fit to data and to compare the different states' characteristics## Code written for 3 states, adapt code to plot models with different number of statescolors <- c("#E69F00", "#56B4E9", "#009E73")mod.new <- mods[[best]]par(mfrow=c(3,1))delta <- as.vector(table(mod.new$states$state)/length(mod.new$states$state))z <- seq(0, max(obs$step, na.rm=T), by=0.5)hist(obs$step,probability=TRUE,breaks=60,col="light grey",border="white",xlab="step length",ylab="density",main="")lines(z,delta[1]*dgamma(z,shape=mod.new$mu.step[1]^2/mod.new$sigma.step[1]^2,scale=mod.new$sigma.step[1]^2/mod.new$mu.step[1]),col=colors[1],lwd=2)lines(z,delta[2]*dgamma(z,shape=mod.new$mu.step[2]^2/mod.new$sigma.step[2]^2,scale=mod.new$sigma.step[2]^2/mod.new$mu.step[2]),col=colors[2],lwd=2)lines(z,delta[3]*dgamma(z,shape=mod.new$mu.step[3]^2/mod.new$sigma.step[3]^2,scale=mod.new$sigma.step[3]^2/mod.new$mu.step[3]),col=colors[3],lwd=2)lines(z,delta[1]*dgamma(z,shape=mod.new$mu.step[1]^2/mod.new$sigma.step[1]^2,scale=mod.new$sigma.step[1]^2/mod.new$mu.step[1]) +        delta[2]*dgamma(z,shape=mod.new$mu.step[2]^2/mod.new$sigma.step[2]^2,scale=mod.new$sigma.step[2]^2/mod.new$mu.step[2]) +        delta[3]*dgamma(z,shape=mod.new$mu.step[3]^2/mod.new$sigma.step[3]^2,scale=mod.new$sigma.step[3]^2/mod.new$mu.step[3]),       lwd=2,lty=2)z <- seq(0, max(obs$divetime, na.rm=T), by=0.5)hist(obs$divetime,probability=TRUE,breaks=60,col="light grey",border="white",xlab="dive duration",ylab="density",main="")lines(z,delta[1]*dgamma(z,shape=mod.new$mu.diveDur[1]^2/mod.new$sigma.diveDur[1]^2,scale=mod.new$sigma.diveDur[1]^2/mod.new$mu.diveDur[1]),col=colors[1],lwd=2)lines(z,delta[2]*dgamma(z,shape=mod.new$mu.diveDur[2]^2/mod.new$sigma.diveDur[2]^2,scale=mod.new$sigma.diveDur[2]^2/mod.new$mu.diveDur[2]),col=colors[2],lwd=2)lines(z,delta[3]*dgamma(z,shape=mod.new$mu.diveDur[3]^2/mod.new$sigma.diveDur[3]^2,scale=mod.new$sigma.diveDur[3]^2/mod.new$mu.diveDur[3]),col=colors[3],lwd=2)lines(z,delta[1]*dgamma(z,shape=mod.new$mu.diveDur[1]^2/mod.new$sigma.diveDur[1]^2,scale=mod.new$sigma.diveDur[1]^2/mod.new$mu.diveDur[1]) +        delta[2]*dgamma(z,shape=mod.new$mu.diveDur[2]^2/mod.new$sigma.diveDur[2]^2,scale=mod.new$sigma.diveDur[2]^2/mod.new$mu.diveDur[2]) +        delta[3]*dgamma(z,shape=mod.new$mu.diveDur[3]^2/mod.new$sigma.diveDur[3]^2,scale=mod.new$sigma.diveDur[3]^2/mod.new$mu.diveDur[3]),       lwd=2,lty=2)z <- seq(0, 1, by=0.001)hist(obs$ODBA,probability=TRUE,breaks=50,col="light grey",border="white",xlab="ODBA",ylab="density",main="",xlim=c(0,0.4))lines(z,delta[1]*dBEINF0(z, mu=mod.new$mu.odba[1], sigma=mod.new$sigma.odba[1], nu=mod.new$nu.odba[1]), col=colors[1],lwd=2)#points(0,delta[1]*dBEINF0(0, mu=mod.new$mu.odba[1], sigma=mod.new$sigma.odba[1], nu=mod.new$nu.odba[1]), col=colors[1],pch=19)lines(z,delta[2]*dBEINF0(z, mu=mod.new$mu.odba[2], sigma=mod.new$sigma.odba[2], nu=mod.new$nu.odba[2]), col=colors[2],lwd=2)#points(0,delta[2]*dBEINF0(0, mu=mod.new$mu.odba[2], sigma=mod.new$sigma.odba[2], nu=mod.new$nu.odba[2]), col=colors[2],pch=19)lines(z,delta[3]*dBEINF0(z, mu=mod.new$mu.odba[3], sigma=mod.new$sigma.odba[3], nu=mod.new$nu.odba[3]), col=colors[3],lwd=2)#points(0,delta[3]*dBEINF0(0, mu=mod.new$mu.odba[3], sigma=mod.new$sigma.odba[3], nu=mod.new$nu.odba[3]), col=colors[3],pch=19)lines(z,delta[1]*dBEINF0(z, mu=mod.new$mu.odba[1], sigma=mod.new$sigma.odba[1], nu=mod.new$nu.odba[1]) +        delta[2]*dBEINF0(z, mu=mod.new$mu.odba[2], sigma=mod.new$sigma.odba[2], nu=mod.new$nu.odba[2]) +        delta[3]*dBEINF0(z, mu=mod.new$mu.odba[3], sigma=mod.new$sigma.odba[3], nu=mod.new$nu.odba[3]),       lwd=2,lty=2)#### 9. Plot the GPS position of dives from the different states on a map to explore geographic patterns of statesmap <- getMap(resolution = "low")par(mfrow=c(1,1))plot(map, xlim=c(-90.6,-90.25), ylim=c(-0.97,-0.75), asp=1, col="darkgray")points(mod.new$states$Longitude, mod.new$states$Latitude, col=colors[mod.new$states$state], type="o", pch=16, cex=0.4)#### 10. Plot boxplots comparing distribution of different dive variables to help interpretation of statesboxplot(mod.new$states$step ~ mod.new$states$state, main="steplength", ylim= c(0, 150), xlab="", cex.lab=0.2)boxplot(mod.new$states$divetime ~ mod.new$states$state, main="Dive_Time", ylim= c(0, 400), xlab="")boxplot(mod.new$states$ODBA ~ mod.new$states$state, main="Time_ODBA", ylim= c(0, 0.5), xlab="")boxplot(mod.new$states$maxdepth ~ mod.new$states$state, main="Depth", ylim= c(0, 250), xlab="")####### Repeat steps 5. to 10. to find the optimal number of states that best fit the data
